# Outlines of the Pathology and Treatment of Syphilis and Allied Diseases

**Published:** 1887-06

**Authors:** 


### Book Reviews.

Outlines of the Pathology and Treatment of Syphilis and Allied Venereal Diseases. *By* Hermann Von Zeissll, M.D., *Late Professor at the Imperial-Royal University of Vienna. Second Edition, revised by* Maximilian Von Zeissll, M.D., Privat-docent, *etc. Authorized edition translated with notes by* H. Raphael, M.D., *Attending Physician for Diseases of the Genito-Urinary Organs, etc. New York:* D. Appleton & Co. *1886. Pp. 402.*

Zeissll's treatise has been so long known and so deservedly well received in its original dress, that an English translation is welcome at once as a valuable addition to the literature of syphilis and allied venereal diseases, in our own tongue. In the present case it may be said that Dr. Raphael has done his work of translation with care and intelligence, and the result is a treatise in the vernacular, and not a clumsy bundling of the author's ideas from one language to another.

It is to be regretted that the revision of the whole, since the appearance of the second edition in 1884, had not rather more fully brought the subject in all its branches up to the date of the translation, though Dr. Raphael's notes supplement this work to a certain extent. For example, in the matter of the gonococcus, which many hold to be the etiological factor in the production of all forms of true gonorrhœa, the present work devotes but a paragraph or two, evidently added by the translator, to the discoveries of Neisser, and the briefest allusion to Bumm's work. In the chapters devoted to consideration of syphilis, the translator has also added a couple of paragraphs merely, devoted to the consideration of the bacilli which Lustgarten and Doutrelepont have regarded as the essential elements of the syphilitic virus. It is here stated that it is "probable that these bacilli actually constitute the syphilitic poison," but one would scarcely conclude from this brief notice that the whole pathology of syphilis is undergoing at the hands of some authors a revision on the basis of what has been written in this connection.

It certainly is well, however, for the practical value of the translation that it has been done by an American. Almost every page bears some fruit of the practical efforts of the American school in the direction of affording the promptest relief to patients actually suffering from the disorders discussed. The conspicuous absence of hints of this character in many German treatises has made the latter unpopular with American readers. For example, there is scarcely a clear distinction made by the German authors between acute and chronic prostatitis, a defect which the translator endeavors to cure by a hint; and, in the treatment of the first-named complication of a blenorrhagia, the application of leeches to the perineum is actually relegated to one of the translator's notes. There are few American practitioners who would, in an acute prostatitis threatening abscess, tie a Nelaton catheter in the urethra, "in order to keep open the canal for the passage of urine," yet this is the first injunction of the German authors for the relief of the most distressing complication of this disorder. However, we can make allowance for a few such minor faults in estimating at its real value the conservative method in which the authors view many methods of treatment and surgical procedures in the disorders of which they treat, that it is easy to see in the course of a few years will be regarded as relics of the brutal period of genito-urinary surgery.

The book is recommended to the American profession as a trustworthy guide in the management of the diseases in the field in which it has so long and so well occupied a high rank.

